# Differential Expression Analysis of Key Inflammatory Mediators in Irreversible Pulpitis for Diagnostic Biomarkers

**DOI:** 10.1016/j.identj.2026.109596

**Published:** 2026-05-31

**Authors:** Raksha Bhat, Sean Prinson D’souza, Preethesh Shetty, Shruthi Padavu, Praveen Rai, Ballamoole Krishna Kumar, Shishir Shetty

**Affiliations:** a Nitte (Deemed to be University), AB Shetty Memorial Institute of Dental Sciences (ABSMIDS), Department of Conservative Dentistry and Endodontics, Mangalore, Karnataka, India; b Nitte (Deemed to be University), Nitte University Centre for Science Education and Research (NUCSER), Department of Infectious Diseases and Microbial Genomics, Mangalore, Karnataka, India

**Keywords:** Biomarkers, Cytokines, Genes, Pulpitis, RNA

## Abstract

**Introduction and Aims:**

The diagnosis of pulpal inflammatory conditions poses significant challenges in clinical endodontics, requiring precise differentiation between reversible and irreversible states. This study investigated the differential expression patterns of 10 inflammatory genes: LCP2, PTPRC, CXCL8, TNFα, IL6, CCL2, MMP9, NOD2, ICAM1, and TLR8 in irreversible pulpitis as potential molecular diagnostic biomarkers.

**Methods:**

In this prospective cross-sectional analytical investigation, pulp tissue samples were collected from 30 subjects with irreversible pulpitis and 10 control subjects with healthy pulp tissue from orthodontically indicated extractions. Clinical diagnosis followed standardized American Association of Endodontists guidelines by two calibrated endodontists (Cohen’s kappa >0.85). RNA isolation utilized TRIzol methodology, followed by quantitative Real-Time PCR analysis with GAPDH normalization and >2-fold change threshold for differential expression.

**Results:**

Analysis revealed varying frequencies of differential gene expression, with CCL2 demonstrating the highest frequency (63.3%, *P* = .001587), followed by MMP9 (60%, *P* = .001453), TNFα and IL6 (56.7% each), CXCL8 and NOD2 (53.3% each), TLR8 (46.7%), ICAM1 and PTPRC (43.3% each), and LCP2 (36.7%). Concurrent differential expression of all 10 markers occurred in 3.3% of cases. Significant correlations were identified between gene expression and clinical parameters, particularly CXCL8 with thermal hyper-response (*r* = 0.641, *P* < .001), TLR8 with thermal hyper-response (*r* = 0.758, *P* < .001) and pain type (*r* = 0.379, *P* = .039), and TNFα with visual analogue scale pain intensity and thermal hyper-response.

**Conclusion:**

Six genes (MMP9, CXCL8, TNFα, IL6, CCL2, NOD2) demonstrated statistically significant upregulation with notable correlations between specific genes and clinical manifestations. These preliminary findings suggest potential utility as diagnostic biomarkers, though clinical implementation requires validation through larger cohort studies.

**Clinical Relevance:**

This molecular profiling approach addresses critical limitations in current endodontic diagnostics with 30% to 40% diagnostic inaccuracies. The identified inflammatory gene signatures provide quantifiable molecular evidence that could enhance clinical decision-making in determining vital pulp therapy vs conventional root canal treatment. Implementation through point-of-care diagnostic platforms may reduce diagnostic uncertainty and facilitate evidence-based endodontic therapy.

## Graphical abstract

**Figure:**
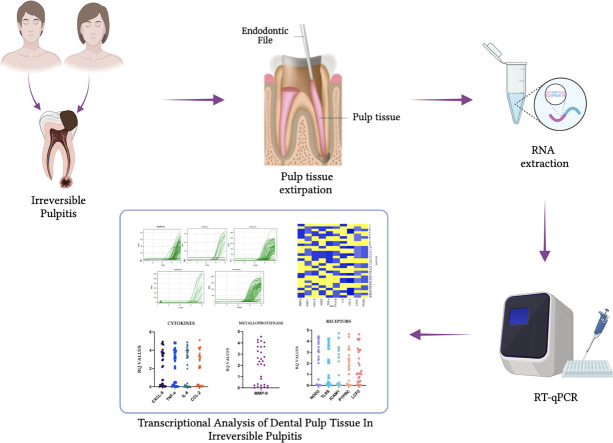

## Introduction

Pulpal inflammatory pathology presents a significant diagnostic challenge in contemporary endodontics, necessitating precise differentiation between reversible and irreversible states for optimal therapeutic intervention. While bacterial infiltration constitutes the primary etiological factor in pulpitis initiation, the pathogenesis involves complex host-pathogen interactions, with host immunological responses significantly modulating disease progression.^1^ The intricate interplay between microbial virulence factors and host defence mechanisms in dental hard tissues manifests through multiple molecular pathways.^2^

Current diagnostic protocols, predicated on AAE guidelines, predominantly rely on clinical symptomatology and pulpal sensitivity testing. However, these methodologies demonstrate inherent limitations in accurately determining inflammatory status.^3^ The discordance between clinical manifestations and histopathological findings, compounded by the subjective nature of pain assessment, underscores the imperative for more objective diagnostic parameters.^4^^,^^5^

The molecular phase of pulpal inflammation is characterized by the upregulation of inflammatory mediators, including cytokines, proteases, growth factors, and antimicrobial peptides, which precede both macroscopic and microscopic tissue alterations.^6^ This temporal sequence presents opportunities for early molecular diagnostic intervention. Contemporary genomic analyses have identified specific inflammatory markers – LCP2, PTPRC, CXCL8, TNFα, IL6, CCL2, MMP9, NOD2, ICAM1, and TLR8 – as potential diagnostic indicators for quantifying inflammatory severity in irreversible pulpitis.^1^, ^2^, ^3^, ^4^, ^5^^,^^7^^,^^8^

The paradigm shift towards pulpal vitality preservation necessitates enhanced diagnostic precision.^5^ Recent advances in molecular detection methodologies have demonstrated the presence of inflammatory mediators in various biological matrices, including gingival crevicular fluid, dentinal fluid, periapical exudates, and pulpal blood.^8^ These developments, in conjunction with emerging nanobiomaterial technologies, suggest promising avenues for improving diagnostic accuracy and treatment outcomes.^9^

The present investigation seeks to elucidate the differential expression patterns of predicted genes in irreversible pulpitis and their correlation with clinical manifestations. This cross-sectional exploratory investigation study aims to establish quantifiable molecular parameters for accurate determination of pulpal inflammatory status, thereby facilitating evidence-based clinical decision-making in minimally invasive pulp preservation protocols. The characterization of these molecular signatures may significantly enhance diagnostic precision and therapeutic outcomes in clinical endodontics.

## Materials and methods

### Study design, participants, and enrolment

A prospective cross-sectional analytical investigation was conducted in strict accordance with the Strengthening the Reporting of Observational Studies in Epidemiology (STROBE) guidelines. The research protocol received institutional ethical committee approval (ABSM/EC/269/2022) and adhered to the principles outlined in the Declaration of Helsinki and Good Clinical Practice guidelines. Written informed consent was obtained from all study participants following comprehensive explanation of the research protocol.

### Patient selection and diagnostic protocol

Following rigorous clinical and radiological screening, 40 subjects (*n* = 30 experimental cohort; *n* = 10 control cohort) within the age range of 18 to 35 years were enrolled based on predetermined inclusion and exclusion parameters. The experimental cohort inclusion criteria mandated; clinical diagnosis of irreversible pulpitis according to standardized American Association of Endodontists guidelines, absence of radiographic periapical pathology, negative history of previous endodontic intervention. Exclusion criteria encompassed; the presence of periodontal pathology (probing depths >3 mm), systemic conditions including but not limited to diabetes mellitus, immunological disorders, and osteoporosis, current pharmacological interventions known to modulate immune response, previous endodontic treatment, presence of internal or external root resorption, history of dental trauma, radiographic evidence of canal calcification, and incomplete root formation. The control cohort comprised vital premolar teeth indicated for orthodontic extraction, specifically excluding specimens with caries, structural anomalies, or compromised vitality.

### Clinical diagnostic methodology

All diagnostic procedures were performed by two calibrated endodontists (Cohen’s kappa >0.85) following a standardized operating protocol. Pulpal diagnosis strictly adhered to AAE guidelines through implementation of: Comprehensive pain history documentation, thermal sensitivity testing utilizing refrigerant spray (Coltène/Whaledent AG) and electric pulp testing (SybronEndo), Percussion and palpation testing, mobility assessment, standardized periapical radiographic examination, pain intensity quantification using 100 mm visual analogue scale (VAS).

### Specimen collection and processing protocol

For the experimental cohort (Irreversible Pulpitis), following administration of local anaesthesia with 2% lidocaine hydrochloride with 1:80,000 epinephrine, strict isolation was achieved using rubber dam with surface disinfection with 70% isopropyl alcohol. Access preparation was performed under 3.5 × magnification utilizing sterile high-speed instrumentation with continuous water coolant. Pulp tissue extraction was accomplished using sterile barbed broaches followed by immediate transfer to cryogenic transport medium maintained at −20°C. Specimens were subsequently stored at −80°C until analysis.

For the control cohort (Healthy Pulp); extracted premolars underwent sequential triple washing with sterile Dulbecco’s phosphate-buffered saline (pH 7.4). Standardized access preparation was performed utilizing Endo Access Bur (Dentsply Sirona) with continuous cooling. Pulp tissue extraction and storage protocols were identical to the experimental cohort methodology.

### Molecular analysis protocol

RNA Extraction and Quantification: Total RNA extraction was performed utilizing TRIzol methodology. Tissue specimens underwent mechanical homogenization in RNAiso plus reagent (Takara Bio Inc), followed by phase separation with chloroform (200 µL, 12,000 × *g*, 15 minutes, 4°C). RNA precipitation was achieved using isopropanol with subsequent 75% ethanol washing steps. Quantitative and qualitative assessment of extracted RNA employed NanoPhotometer spectrophotometry (Implen GmbH) with analysis of A260/A280 ratios.

Real-time PCR Analysis: Quantitative PCR analysis was performed using 1 µg DNase-digested RNA templates. Expression profiles of target genes; LCP2, PTPRC, CXCL8, TNFα, IL6, CCL2, MMP9, NOD2, ICAM1, and TLR8, were analysed using CFX Opus 96 Real-Time PCR System (Bio-Rad Laboratories) with One-Step TB Green PrimeScript RT-PCR Kit II (Takara Bio Inc). Reaction conditions were standardized as follows: initial denaturation (95°C, 30 seconds), followed by 40 cycles of denaturation (95°C, 5 seconds), annealing (60°C, 30 seconds), and extension (72°C, 30 seconds). Gene expression normalization employed GAPDH as endogenous reference, with relative quantification calculated via 2^^−ΔΔ^*^Ct^* methodology. Differential expression threshold was established at >2-fold change relative to controls, with all analyses performed in technical triplicates.

### Statistical analysis

Statistical analyses were performed using GraphPad Prism version 9.0 (GraphPad Software). Relative gene expression data were presented as mean ± standard deviation (SD) of fold changes. Gene expression data from the irreversible pulpitis group (*n* = 30) were compared to the control group baseline (normalized expression value of 1.0, representing healthy pulp tissue, *n* = 10). One-sample *t* tests were performed to compare the mean fold-change values in the pulpitis group against the control baseline of 1.0. Additionally, Wilcoxon signed-rank tests were conducted as nonparametric alternatives. Statistical significance was set at *P* < .05. Descriptive statistics include mean ± SD, median with interquartile range (IQR), and range. Correlation analyses were performed to examine relationships between gene expression levels and clinical parameters. Spearman’s rank correlation coefficient was calculated for associations between fold-change values and continuous variables (pain intensity on VAS, symptom duration in weeks). Point-biserial correlation coefficients were calculated for associations with binary variables (thermal hyper response: present vs absent; pain type: continuous vs intermittent). Statistical significance was set at *P* < .05 for all correlation analyses.

## Results

### Clinical and diagnostic parameters

The clinical investigation encompassed 30 cases of diagnosed irreversible pulpitis, with all cases presenting in mandibular first molars – specifically tooth numbers 36 (mandibular left first molar) and 46 (mandibular right first molar). The pain intensity reported by patients ranged from moderate to severe (VAS scores 5-9), with a mean pain score of 6.9. The duration of symptoms varied from 1 week to 3 months, with the majority of cases (43.3%) reporting symptoms for 2 to 4 weeks. The predominant pain characteristics included continuous pain (33.3%), dull pain (30%), and intermittent pain (23.3%), with some cases reporting specific aggravating factors such as food lodgement and positional changes. All cases demonstrated positive response to percussion testing and thermal stimulation, with 36.7% exhibiting hyper response to thermal testing. Radiographic examination consistently revealed carious lesions with radiolucency involving enamel and dentin approaching the pulp, with 13.3% of cases showing mild widening of the lamina dura. The distribution between right and left molars was relatively balanced, with 17 cases (56.7%) involving. tooth 46 and 13 cases (43.3%) involving tooth 36. The consistent clinical and radiographic findings across all cases supported the definitive diagnosis of chronic irreversible pulpitis. The complete diagnostic findings and clinical parameters for the study cohort are presented in Table 1

**Table 1:** Comprehensive analysis of case history and diagnostic tests of the cases selected for the study.

Case no	Chief complaint	Pain quality			Diagnostic tests				Final diagnosis
		Quality and intensity	Duration	Pain scale	Tooth involved	Percussion	Radiographic diagnosis	Thermal test	
1	Pain in the lower right back region	Continuous pain	2 mo	6	46	Positive	Radiolucency involving the enamel, dentin approaching the pulp.	Positive (Hyper response)	Chronic irreversible pulpitis on 46.
2	Pain in the lower left back tooth region, especially on lying down	Lingering pain	1 mo	8	36	Positive	Radiolucency involving the enamel, dentin approaching the pulp.	Positive	Chronic irreversible pulpitis on 36.
3	Pain in the lower left back region	Throbbing continuous pain	2 wk	6	36	Positive	Large radiolucency through the enamel, dentin, and the pulp. Mild widening of the lamina dura seen.	Positive (Hyper response)	Chronic irreversible pulpitis on 36.
4	Pain in the lower right back region	Dull pain	1 mo	8	46	Positive	Radiolucency involving the enamel, dentin approaching the pulp.	Positive	Chronic irreversible pulpitis on 46.
5	Food lodgement and pain in the lower right back region	Dull throbbing pain	3 mo	7	46	Positive	Proximal caries involving the enamel, dentin approaching the pulp.	Positive	Chronic irreversible pulpitis on 46.
6	Pain in the lower left back region accompanied with food impaction	Continuous pain	1 wk	6	36	Positive	Radiolucency in the proximal region involving the enamel, dentin approaching the pulp.	Positive (Hyper response)	Chronic irreversible pulpitis on 36.
7	Pain in the lower right back region	Spontaneous pain	3 wk	7	46	Positive	Radiolucency involving the enamel, dentin approaching the pulp.	Positive (Hyper response)	Chronic irreversible pulpitis on 46.
8	Pain in the lower right back region	Food lodgement causing continuous pain.	1 wk	9	46	Positive	Radiolucency involving the enamel, dentin approaching the pulp.	Positive	Chronic irreversible pulpitis on 46.
9	Pain in the lower right back region	Dull pain	2 wk	8	46	Positive	Radiolucency involving the enamel, dentin approaching the pulp.	Positive	Chronic irreversible pulpitis on 46.
10	Pain in the lower left back region	Continuous pain on lying down.	2 wk	7	36	Positive	Radiolucency involving the enamel, dentin approaching the pulp.	Positive (Hyper response)	Chronic irreversible pulpitis on 36.
11	Pain in the lower right back region	Continuous pain on chewing	1 mo	5	46	Positive	Radiolucency involving the enamel, dentin approaching the pulp.	Positive (Hyper response)	Chronic irreversible pulpitis on 46.
12	Pain in the lower left back region	Dull pain	3 wk	5	36	Positive	Radiolucency involving the enamel, dentin approaching the pulp.	Positive	Chronic irreversible pulpitis on 36.
13	Pain in the lower right back region	Dull throbbing continuous pain	2 mo	8	46	Positive	Radiolucency involving the enamel, dentin approaching the pulp. Mild widening of the lamina dura seen.	Positive	Chronic irreversible pulpitis on 46.
14	Pain in the lower left back region	Dull pain	1 mo	7	36	Positive	Radiolucency involving the enamel, dentin approaching the pulp.	Positive	Chronic irreversible pulpitis on 36.
15	Pain in the lower right back region	Continuous pain on food lodgement	1 wk	7	46	Positive	Radiolucency involving the enamel, dentin approaching the pulp.	Positive	Chronic irreversible pulpitis on 46.
16	Pain in the lower left back region	Dull continuous pain	2 wk	8	36	Positive	Radiolucency involving the enamel, dentin approaching the pulp.	Positive	Chronic irreversible pulpitis on 36.
17	Pain in the lower right back region	Continuous pain	2 wk	7	46	Positive	Radiolucency involving the enamel, dentin approaching the pulp.	Positive (Hyper response)	Chronic irreversible pulpitis on 46.
18	Pain in the lower left back region	Dull intermittent pain	1 mo	8	36	Positive	Radiolucency involving the enamel, dentin approaching the pulp. Mild widening of the lamina dura seen.	Positive	Chronic irreversible pulpitis on 36.
19	Pain in the lower right back region	Mild continuous pain	2 wk	7	46	Positive	Radiolucency involving the enamel, dentin approaching the pulp.	Positive (Hyper response)	Chronic irreversible pulpitis on 46.
20	Pain in the lower right back region	Dull continuous pain	1 wk	6	46	Positive	Radiolucency involving the enamel, dentin approaching the pulp.	Positive	Chronic irreversible pulpitis on 46.
21	Pain in the lower left back region	Dull mild intermittent pain	1 mo	5	46	Positive	Radiolucency involving the enamel, dentin approaching the pulp.	Positive	Chronic irreversible pulpitis on 46.
22	Pain in the lower right back region	Intermittent pain	1 mo	8	46	Positive	Radiolucency involving the enamel, dentin approaching the pulp. Mild widening of the lamina dura seen.	Positive	Chronic irreversible pulpitis on 46.
23	Pain in the lower left back region	Mild Continuous pain	3 wk	7	36	Positive	Radiolucency involving the enamel, dentin approaching the pulp.	Positive (Hyper response)	Chronic irreversible pulpitis on 36.
24	Pain in the lower right back region	Continuous pain	1 wk	6	46	Positive	Radiolucency involving the enamel, dentin approaching the pulp.	Positive (Hyper response)	Chronic irreversible pulpitis on 46.
25	Pain in the lower left back region	Mild intermittent dull pain	3 wk	6	36	Positive	Radiolucency involving the enamel, dentin approaching the pulp.	Positive	Chronic irreversible pulpitis on 36.
26	Pain in the lower right back region	Continuous dull pain	2 mo	6	46	Positive	Radiolucency involving the enamel, dentin approaching the pulp.	Positive	Chronic irreversible pulpitis on 46.
27	Pain in the lower right back region	Continuous dull pain	1 mo	7	46	Positive	Radiolucency involving the enamel, dentin approaching the pulp.	Positive	Chronic irreversible pulpitis on 46.
28	Pain in the lower left back region	Mild intermittent pain	3 wk	8	46	Positive	Proximal radiolucency involving the enamel, dentin approaching the pulp.	Positive	Chronic irreversible pulpitis on 46.
29	Pain in the lower left back region	Intermittent dull pain	2 wk	6	46	Positive	Radiolucency involving the enamel, dentin approaching the pulp.	Positive	Chronic irreversible pulpitis on 36.
30	Pain in the lower left back region	Dull intermittent pain	1 mo	7	36	Positive	Radiolucency involving the enamel, dentin approaching the pulp.	Positive	Chronic irreversible pulpitis on 36.

### Molecular expression analysis and statistical comparisons

**Fig. 1:**
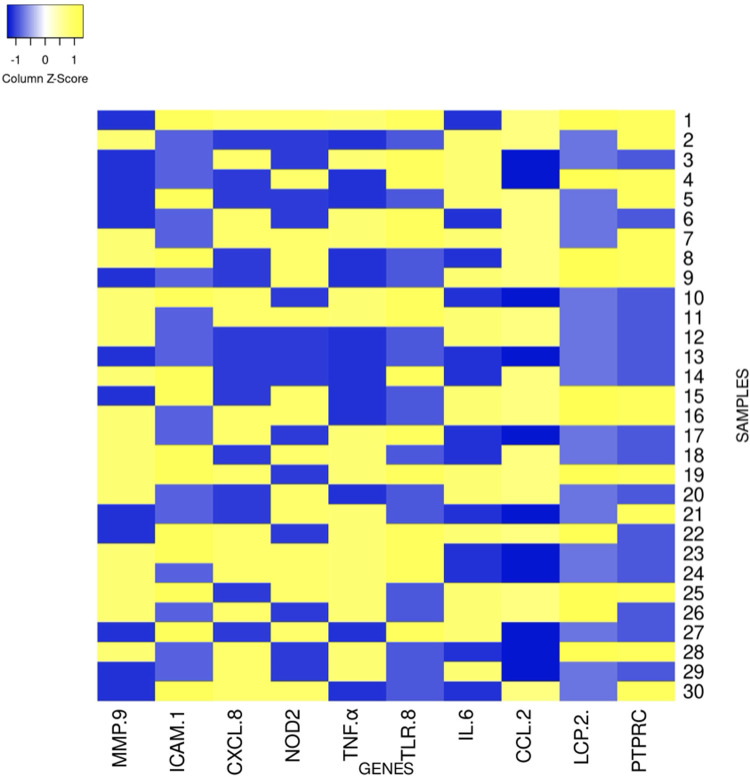
Comprehensive analysis of the relative quantification (RQ) values for the differentially expressed inflammatory genes in irreversible pulpitis.

Quantitative PCR analysis generated characteristic amplification curves demonstrating sigmoid patterns when plotting fluorescent signal intensity against cycle number (Figure 1). Differential gene expression was determined using the 2^^−ΔΔ^*^Ct^* methodology, with a predetermined threshold of greater than twofold change in expression relative to control specimens.

Statistical comparison of gene expression between the irreversible pulpitis group (*n* = 30) and control group (*n* = 10) revealed significant differential expression across the inflammatory marker panel (Tables 2 and 3). One-sample *t* tests comparing mean fold-change values against the control baseline (normalized expression of 1.0) demonstrated that eight of 10 genes (80%) showed statistically significant upregulation (*P* < .05). CCL2 exhibited the highest frequency of differential expression, with upregulation detected in 19 specimens (63.3%, mean fold-change: 2.10 ± 1.72, *P* = .002), followed by MMP9 in 18 specimens (60.0%, mean: 2.03 ± 1.60, *P* = .001). TNFα and IL6 showed parallel expression patterns, each demonstrating upregulation in 17 specimens (56.7%, mean: 2.13 ± 1.83 and 2.15 ± 1.92-fold, respectively, both *P* < .01). CXCL8 and NOD2 exhibited identical detection frequencies of 16 specimens each (53.3%, mean: 2.05 ± 1.86, *P* = .005 and 1.83 ± 1.78, *P* = .016, respectively). TLR8 demonstrated moderate detection frequency with 14 specimens (46.7%, mean: 1.60 ± 1.62, *P* = .052), while ICAM1 and PTPRC were differentially expressed in 13 specimens each (43.3%, mean: 1.56 ± 1.76, *P* = .091 and 1.73 ± 1.49, *P* = .012, respectively). LCP2 demonstrated the lowest frequency of differential expression, being upregulated in 11 specimens (36.7%, mean: 1.63 ± 1.52, *P* = .031). Notably, simultaneous differential expression of all 10 inflammatory markers was observed in a single specimen (3.3%).

**Table 2:** Relative gene expression of the relative quantification (RQ) values for the differentially expressed inflammatory genes in irreversible pulpitis.

Target gene	MMP-9	ICAM-1	CXCL-8	NOD2	TNF-α	TLR-8	IL-6	CCL-2	LCP-2	PTPRC
Sample 1	1	2.58	3.45	2.36	2.82	3.31	0.22	2.82	3.22	4.38
Sample 2	2.76	0.01	0.35	0.08	0.45	0.9	2.43	3.6	0	3.23
Sample 3	0.29	0	3.66	0.02	4.22	4.03	4.04	0	0.1	0
Sample 4	0.81	0.05	0.01	3.45	0.02	2.223	3.23	0.03	4.21	2.42
Sample 5	2.34	3.27	0.13	0.04	0.2	0.1	4.01	4.23	0.02	4.64
Sample 6	0.13	0.1	2.16	0.01	3.11	3.54	0	2.01	0.01	0
Sample 7	3.67	0.03	4.93	2.02	4.81	2.09	3.43	2.2	0.36	2.45
Sample 8	4.28	4.72	0.11	4.345	0.02	0.07	0.2	2.06	3.34	2.23
Sample 9	0.09	0	0.08	2.5967	0.3	0.2	4.08	4.3	2.01	0
Sample 10	4.18	4.32	3.778	0	2.21	3.72	0	0.21	0	0.06
Sample 11	2.09	0.25	4.74	4.03	3.78	3.56	3.53	3.23	0.3	0.162
Sample 12	2.12	0	0	0.05	0.46	0	3.87	4.2	1.3	1.86
Sample 13	0.04	0.05	0.05	0.04	0.49	0	0.1	0	1.82	1.02
Sample 14	2.02	3.45	0.28	0.54	0	3.92	0.4	5.06	1.09	0
Sample 15	0.23	3.28	0.01	3.02	0.51	0.22	4.65	3.2	4.62	2.35
Sample 16	3.64	0.23	3.34	4.43	0	0.1	3.77	4.2	2.44	3.27
Sample 17	4.06	0	3.51	0	3.45	4.23	0.04	0.01	1	1.5
Sample 18	3.16	4.71	0.76	3.87	4.02	0	0.01	2.23	0.41	1
Sample 19	2.56	2.17	2.98	0	4.82	2.09	4.53	3.4	3.18	3.53
Sample 20	2.67	0.07	0.06	2.3	0.03	0	4.03	4.03	0.67	1.65
Sample 21	0.18	0.92	0	3.02	3.93	0.65	0	0.1	1.11	2.73
Sample 22	0.02	2.51	4.72	0.06	3.72	2.01	4.92	3.02	4.11	1.77
Sample 23	2.38	3.46	3.81	4.278	4.07	4.23	0.07	0	1.05	0.02
Sample 24	4.56	0.1	4.05	3.76	3.22	2.32	0.04	0.2	0.32	0.43
Sample 25	3.27	4.27	0.72	4.231	4.77	0.68	4.32	2.9	3.21	4.41
Sample 26	4.04	0.12	2.9	0.01	3.23	0	3.34	2.08	4.22	0.05
Sample 27	0.22	2.82	0.21	3.21	0.07	3.3	2.02	0.03	0.77	0
Sample 28	3.67	0.04	2.28	0.04	2.03	0.33	0	0.1	3.02	3.34
Sample 29	0.34	0.2	3.67	0	3.08	0.1	3.2	0.09	1	1.4
Sample 30	0.02	3.12	4.62	3.07	0.09	0.12	0.04	3.348	0	2.08

**Table 3:** Descriptive statistics of gene expression in irreversible pulpitis.

Gene	Mean ± SD	Median	IQR (Q1-Q3)	Range	Samples >2-fold	% >2-fold	*P* value†
MMP9	2.03 ± 1.60	2.23	0.24-3.55	0.02-4.56	18/30	60.0%	.001453⁎⁎
ICAM1	1.56 ± 1.76	0.24	0.05-3.23	0.00-4.72	13/30	43.3%	.091210
CXCL8	2.05 ± 1.86	2.22	0.12-3.67	0.00-4.93	16/30	53.3%	.004599⁎⁎
NOD2	1.83 ± 1.78	2.16	0.04-3.39	0.00-4.43	16/30	53.3%	.016056⁎
TNFα	2.13 ± 1.83	2.51	0.23-3.76	0.00-4.82	17/30	56.7%	.002036⁎⁎
TLR8	1.60 ± 1.62	0.79	0.10-3.31	0.00-4.23	14/30	46.7%	.051649
IL6	2.15 ± 1.92	2.82	0.05-3.97	0.00-4.92	17/30	56.7%	.002699⁎⁎
CCL2	2.10 ± 1.72	2.21	0.10-3.39	0.00-5.06	19/30	63.3%	.001587⁎⁎
LCP2	1.63 ± 1.52	1.07	0.33-3.14	0.00-4.62	11/30	36.7%	.030898⁎
PTPRC	1.73 ± 1.49	1.71	0.09-2.66	0.00-4.64	13/30	43.3%	.011843⁎

⁎ *P* < 0.05.

⁎⁎ *P* < 0.01.

† One-sample *t*-test comparing pulpitis group mean against control baseline of 1.0.

### Expression pattern characterization

Comprehensive analysis of relative quantification (RQ) values revealed distinct molecular signatures characterized by substantial intersample heterogeneity (Table 4). The expression patterns across all 10 inflammatory genes demonstrated marked variability, as evidenced by high SDs and wide IQRs (Table 2). MMP9, a matrix metalloproteinase involved in extracellular matrix degradation, exhibited expression ranging from 0.02 to 4.56-fold (median: 2.23, IQR: 0.25-3.55). Proinflammatory cytokines TNFα and IL6 displayed similarly heterogeneous patterns, with TNFα ranging from 0.00 to 4.82-fold (median: 2.52, IQR: 0.23-3.77) and IL6 from 0.00 to 4.92-fold (median: 2.82, IQR: 0.05-3.98). The chemokines CXCL8 and CCL2 demonstrated comparable variability, with CXCL8 ranging from 0.00 to 4.93-fold (median: 2.22, IQR: 0.12-3.67) and CCL2 from 0.00 to 5.06-fold (median: 2.22, IQR: 0.10-3.39). Pattern recognition receptors NOD2 and TLR8 exhibited differential activation profiles across samples, with NOD2 ranging from 0.00 to 4.43-fold (median: 2.16, IQR: 0.04-3.39) and TLR8 from 0.00 to 4.23-fold (median: 0.79, IQR: 0.10-3.31), potentially reflecting variable microbial burden or immune activation states. The adhesion molecule ICAM1 demonstrated expression from 0.00 to 4.72-fold (median: 0.24, IQR: 0.05-3.23), possibly indicating varying degrees of leukocyte recruitment and infiltration. Lymphocyte-associated markers LCP2 and PTPRC showed expression ranges of 0.00 to 4.62-fold (median: 1.07, IQR: 0.33-3.14) and 0.00 to 4.64-fold (median: 1.71, IQR: 0.09-2.66), respectively, suggesting variable lymphocytic involvement in the inflammatory process.

**Table 4:** Statistical comparison of mean gene expression between irreversible pulpitis and control groups.

Gene	Control group (*n* = 10)	Pulpitis group (*n* = 30)	*t*-statistic	*P* value (*t* test)	Significance
MMP9	1.00 ± 0.20	2.03 ± 1.60	3.518	.001453	⁎⁎
ICAM1	1.00 ± 0.20	1.56 ± 1.76	1.747	.091210	ns
CXCL8	1.00 ± 0.20	2.05 ± 1.86	3.071	.004599	⁎⁎
NOD2	1.00 ± 0.20	1.83 ± 1.78	2.557	.016056	⁎
TNFα	1.00 ± 0.20	2.13 ± 1.83	3.389	.002036	⁎⁎
TLR8	1.00 ± 0.20	1.60 ± 1.62	2.030	.051649	ns
IL6	1.00 ± 0.20	2.15 ± 1.92	3.281	.002699	⁎⁎
CCL2	1.00 ± 0.20	2.10 ± 1.72	3.485	.001587	⁎⁎
LCP2	1.00 ± 0.20	1.63 ± 1.52	2.269	.030898	⁎
PTPRC	1.00 ± 0.20	1.73 ± 1.49	2.686	.011843	⁎

Control group represents healthy pulp tissue with normalised expression ≈1.0 (reference baseline after GAPDH normalisation).

⁎ *p* < 0.05 (significant); ns, not significant (*p* ≥ 0.05).

⁎⁎ *p* < 0.01 (very significant).

Correlation analyses revealed specific associations between gene expression patterns and clinical parameters (Table 5). Thermal hyper-response demonstrated the strongest correlations, with TLR8 expression showing a strong positive association (*r* = 0.758, *P* < .001), followed by CXCL8 (*r* = 0.641, *P* < .001) and TNFα (*r* = 0.598, *P* < .001). TNFα expression exhibited a moderate negative correlation with pain intensity (Spearman’s *ρ* = −0.373, *P* = .042). This statistical association suggests an inverse relationship between TNFα expression levels and self-reported pain scores; however, the cross-sectional nature of this study precludes determination of causality or directionality. TLR8 expression positively correlated with continuous-type pain patterns (*r* = 0.379, *P* = .039). No significant correlations were observed between gene expression and symptom duration (all *P* > .05). These associations between specific inflammatory markers and clinical manifestations support their potential utility as diagnostic biomarkers, though the cross-sectional design limits causal inference.

**Table 5:** Correlation analysis between gene expression and clinical parameters.

Gene	VAS pain *ρ*	*P* value	Duration *ρ*	*P* value	Thermal *r*	*P* value	Pain type *r*	*P* value
MMP9	0.057	.7633	−0.274	.1428	0.209	.2688	0.272	.1455
ICAM1	0.102	.5916	0.114	.5471	−0.106	.5755	−0.149	.4309
CXCL8	−0.106	.5782	−0.048	.8005	0.641	.0001⁎⁎	0.270	.1489
NOD2	0.161	.3949	0.010	.9570	−0.073	.6995	0.038	.8405
TNFα	−0.373	.0424⁎	−0.027	.8867	0.598	.0005⁎⁎	0.112	.5565
TLR8	−0.171	.3657	−0.102	.5907	0.758	.0000⁎⁎	0.379	.0390⁎
IL6	−0.036	.8520	−0.063	.7406	−0.210	.2656	−0.082	.6672
CCL2	0.027	.8880	0.047	.8069	−0.287	.1237	−0.179	.3444
LCP2	0.215	.2549	0.044	.8172	−0.320	.0851	−0.088	.6453
PTPRC	0.130	.4926	0.166	.3814	−0.231	.2198	−0.225	.2320

VAS pain: Pain intensity (visual analogue scale, 0-10); Duration: Symptom duration in weeks; Thermal: Thermal hyper-response (present/absent); Pain type: Continuous vs intermittent pain; ρ, Spearman's rank correlation coefficient; *r*, point-biserial correlation coefficient.

⁎ *P* < 0.05.

⁎⁎ *P* < 0.001.

**Fig. 2:**
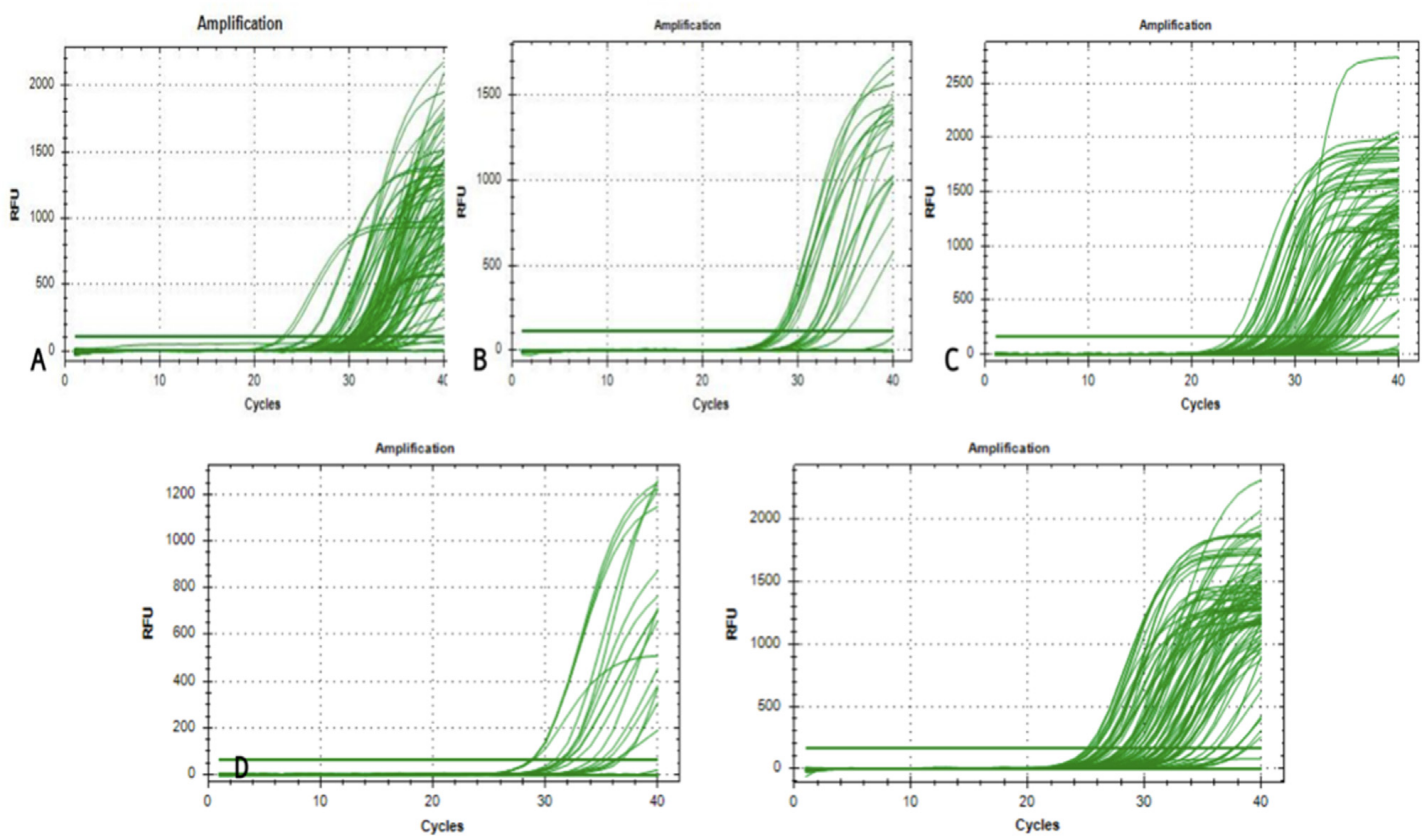
Graphs representing the real-time PCR Amplification plot for the target genes in part (A) – healthy pulp tissue samples and parts (B-E) – chronic irreversible pulpitis tissue samples.

**Fig. 3:**
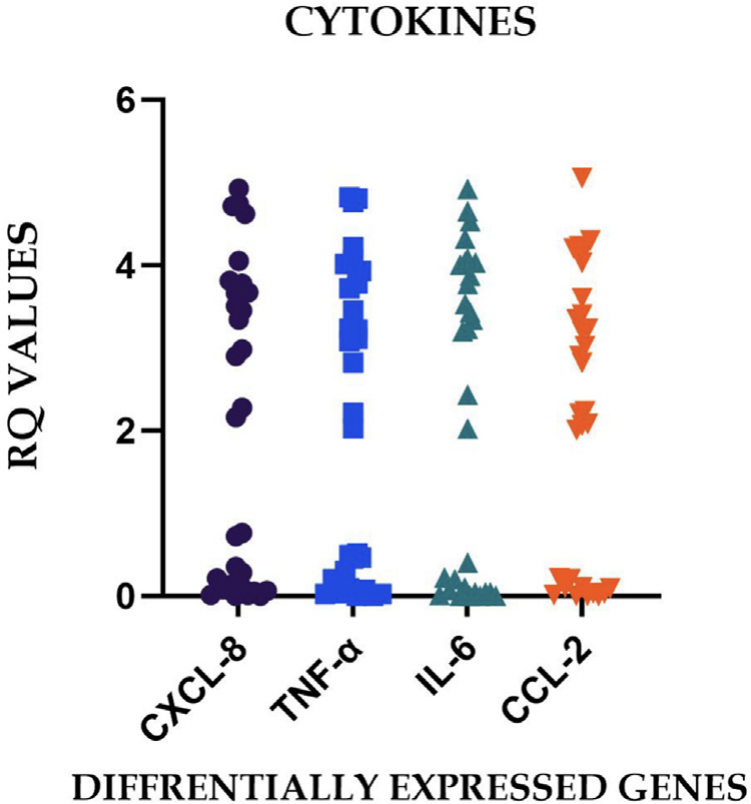
Relative gene expression of the relative quantification (RQ) values for the differentially expressed cytokines in irreversible pulpitis.

**Fig. 4:**
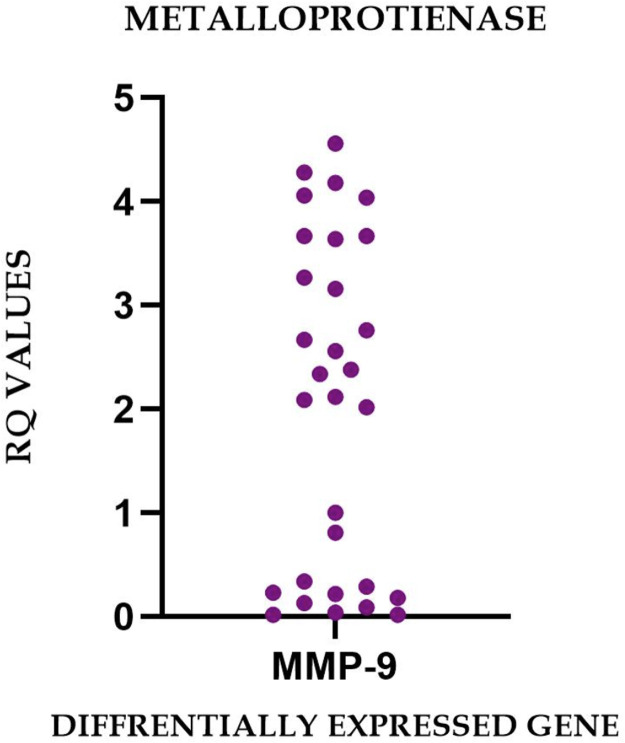
Relative gene expression of the relative quantification (RQ) values for the differentially expressed metalloprotienases in irreversible pulpitis.

**Fig. 5:**
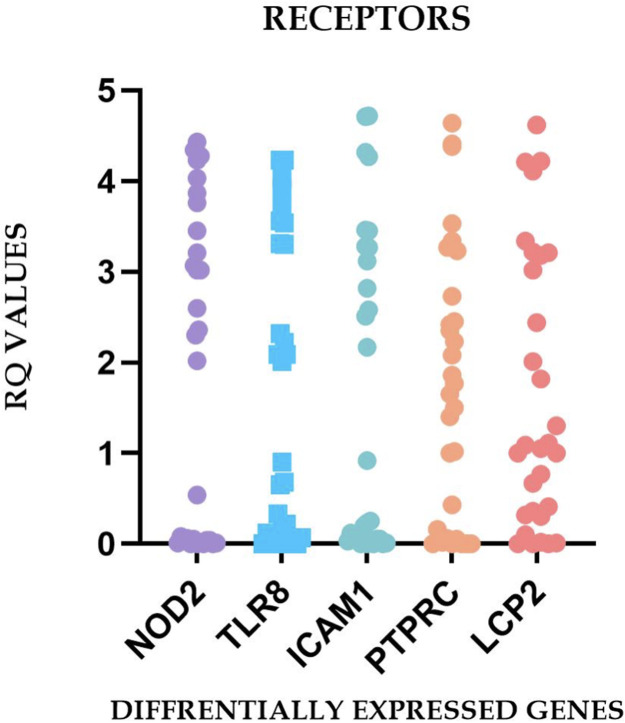
Relative gene expression of the relative quantification (RQ) values for the differentially expressed receptors in irreversible pulpitis.

The observed heterogeneity in gene expression profiles, reflected in the substantial SDs and broad expression ranges (Table 2), indicates that irreversible pulpitis manifests diverse molecular phenotypes across patients. This variability may represent different stages of inflammatory progression, varying microbial challenges, individual host response patterns, or a combination of these factors. The differential expression patterns observed across the sample cohort are graphically represented in Figures 2 to 5, illustrating the characteristic molecular profiles associated with irreversible pulpitis.

## Discussion

The molecular pathobiology of pulpal inflammation, elucidated through comprehensive gene expression analysis in the present investigation, reveals intricate and interconnected networks of inflammatory mediators and signalling cascades that fundamentally alter our understanding of irreversible pulpitis. Integration of our findings with existing literature provides unprecedented insights into the complex mechanisms underlying pulpal inflammatory processes and their potential diagnostic implications.

The correlation analyses revealed clinically meaningful associations between specific inflammatory markers and objective clinical parameters. The strong positive correlations between thermal hyper-response and TLR8, CXCL8, and TNFα expression (*r* = 0.758, 0.641, and 0.598, respectively, all *P* < .001) indicate significant associations between these inflammatory markers and thermal sensitivity. These findings are consistent with the hypothesis that pattern recognition receptors and proinflammatory cytokines may contribute to neural sensitization processes underlying heightened thermal sensitivity in irreversible pulpitis. However, the cross-sectional design precludes determination of causal relationships, and mechanistic validation studies are required to establish causality. The TLR8-mediated activation of innate immunity may contribute to neurogenic inflammation through cytokine-induced sensitization of nociceptors, consistent with previous findings by Khorasani et al. The unexpected negative correlation between TNFα expression and pain intensity (*ρ* = −0.373, *P* = .042) warrants further investigation, as it contrasts with the conventional understanding of TNFα as a primary mediator of inflammatory pain. This finding may reflect complex regulatory mechanisms or temporal dynamics in cytokine-mediated pain modulation, where sustained TNFα elevation could potentially trigger compensatory anti-inflammatory or analgesic pathways. However, the cross-sectional nature of this study limits interpretation of temporal relationships, and mechanistic studies are needed to determine whether this represents a causal relationship or a consequence of other physiological processes. The absence of significant correlations with symptom duration indicates that the molecular inflammatory profile is not associated with disease chronicity in this cross-sectional analysis, but rather may represent individual variations in host immune responses. These associations demonstrate the complexity of pulpal inflammation and provide preliminary evidence supporting the development of multimarker diagnostic approaches that integrate molecular profiles with clinical presentations for improved diagnostic accuracy, pending validation in prospective studies.

The present observation of elevated MMP9 expression in 60% of irreversible pulpitis specimens significantly extends the current understanding of proteolytic activity in pulpal inflammation. This finding substantiates and expands upon seminal documentation of enhanced gelatinolytic activity in inflamed pulp tissue by Hannas et al.^10^ The detection of MMP9 upregulation aligns with the quantification of elevated MMP9 levels using sophisticated microcapillary sampling techniques by Mente et al.^11^ However, as demonstrated in the comprehensive analysis by Brizuela et al,^12^ our findings support the critical conclusion that MMP9 expression alone lacks sufficient specificity to definitively distinguish between reversible and irreversible pulpitis, thus necessitating a more sophisticated multimarker diagnostic approach.

The concurrent elevation of ICAM1 expression, observed in 43.3% of specimens, provides molecular validation and mechanistic insight into the findings regarding the role of ICAM1 in pulpal inflammation by De Rossi et al.^13^ This expression pattern is consistent with an integral role of ICAM1 in the inflammatory cascade, particularly in facilitating leukocyte adhesion and transmigration across endothelial barriers during the inflammatory response. The current detailed analysis of cytokine expression patterns reveals significant upregulation of IL6, IL8, and TNFα (56.7% differential expression), providing comprehensive molecular context to the observations of Abd-Elmeguid et al^14^ while offering additional insights through concurrent analysis of multiple inflammatory mediators.

The present observation of NOD2 upregulation (53.3%) provides molecular confirmation of findings regarding NOD2 expression in human dental pulp tissue stated by Keller et al.^15^ It is consistent with a sophisticated mechanistic link between TLR2 ligand-induced NOD2 upregulation and odontoblast-mediated host defines against bacterial invasion. The elevated PTPRC expression patterns align with the observations of Zhou et al,^16^ while our concurrent analysis of multiple inflammatory mediators provides unprecedented insights into its role within the broader inflammatory network.

A particularly significant contribution of the present investigation is the demonstration of highest frequency of differential expression of CCL2 (63.3%). This finding substantially extends observations of elevated CCL2 expression in chronic periapical lesions by Tavares et al and Hui et al.^17^^,^^18^ The consistent upregulation of CCL2 suggests its potential utility as a key diagnostic marker for pulpal inflammation and provides crucial insights into chemokine-mediated inflammatory processes in pulpal pathology. The observed LCP2 upregulation, though showing the lowest frequency (36.7%), represents the first comprehensive demonstration of its concurrent expression with other inflammatory mediators in irreversible pulpitis, suggesting its potential role in the inflammatory cascade and signal transduction pathways.

Also, the current investigation revealed previously unrecognized correlations between patient-reported pain severity and specific gene expression patterns. The unusual finding of lower expression of IL8, TNFα, and TLR8 in specimens from patients experiencing mild pain compared to those with severe pain indicates a more complex association between molecular markers and clinical symptoms than previously recognized in the literature.^19^ This observation challenges existing paradigms and suggests the need for a more sophisticated understanding of the molecular basis of pulpitis-associated pain.

While the findings successfully demonstrate significant molecular distinctions between irreversible pulpitis and healthy pulpal tissue, establishing the diagnostic specificity of these biomarkers requires validation against reversible pulpitis cases. The intermediate inflammatory state of reversible pulpitis, characterized by its potential for recovery, represents a critical diagnostic challenge in clinical practice. However, obtaining confirmed reversible pulpitis samples presents methodological complexities, as definitive histological validation would require tooth extraction, thereby precluding preservation of pulpal vitality. Future investigations should incorporate reversible pulpitis samples using refined clinical diagnostic criteria and explore whether the identified inflammatory markers demonstrate differential expression patterns that can reliably distinguish between reversible and irreversible inflammatory states, thereby enhancing clinical decision-making regarding vital pulp therapy vs conventional root canal treatment. The sample size represents another limitation of this investigation. While the irreversible pulpitis group (*n* = 30) provided adequate statistical power to detect significant differential expression across multiple genes, the control group (*n* = 10) was constrained by the practical and ethical challenges of obtaining healthy pulp tissue from sound teeth undergoing orthodontically indicated extractions. The modest control group size may limit the precision of baseline expression estimates and potentially affect the generalizability of our findings to broader populations. Despite demonstrating statistically significant differences for 8 of 10 inflammatory markers, validation studies with larger, multicentre cohorts are essential to confirm these molecular signatures, establish clinically applicable diagnostic thresholds, and assess the reproducibility of these findings across diverse patient populations and clinical settings. Additionally, larger sample sizes would enable more sophisticated stratified analyses examining potential confounding variables such as age, sex, tooth type, and extent of carious lesion, which may influence inflammatory gene expression patterns.

As a cross-sectional exploratory study, this investigation lacks an independent validation cohort, representing a significant limitation. Furthermore, the cross-sectional design of this study limits causal inference, as observed correlations between gene expression and clinical parameters represent statistical associations that cannot establish directionality or mechanistic causality. Longitudinal studies and functional validation experiments are necessary to determine whether these molecular signatures causally influence clinical manifestations or represent downstream consequences of the inflammatory process. The absence of external validation limits our ability to assess the generalizability, reproducibility, and clinical utility of these molecular signatures across different clinical settings and patient demographics. Prospective validation studies are essential to confirm these findings, establish standardized diagnostic thresholds with defined sensitivity and specificity values, evaluate interlaboratory reproducibility of gene expression measurements, and assess the clinical utility of these biomarkers in guiding treatment decisions. Future investigations should employ multicentre designs with geographically and demographically diverse cohorts, utilize standardized specimen collection and processing protocols, and incorporate longitudinal follow-up to correlate molecular profiles with treatment outcomes. Additionally, validation studies should examine the performance of these biomarkers in distinguishing not only irreversible pulpitis from healthy tissue but also from reversible pulpitis and other pulpal pathologies, thereby establishing their diagnostic specificity and clinical applicability.

These comprehensive findings collectively advance our understanding of pulpal pathophysiology by demonstrating the concurrent activation of multiple inflammatory pathways. While individual markers have shown promise in previous studies, our detailed analysis suggests that accurate diagnosis of irreversible pulpitis requires evaluation of multiple molecular markers in combination.^20^^,^^21^ This integrated approach could potentially enhance diagnostic accuracy and guide the development of targeted therapeutic interventions in clinical endodontics.

The validation of these molecular signatures in larger cohorts across various stages of chronic irreversible pulpitis remains essential before clinical implementation. Furthermore, the development of practical, chair-side diagnostic tools based on these molecular markers presents significant technical challenges that must be addressed systematically. The correlation between molecular signatures and treatment outcomes requires further investigation to establish predictive biomarkers for pulpal healing potential. The elucidation of these molecular networks provides a robust framework for future investigations in pulpal pathobiology. This enhanced understanding may facilitate the development of more precise diagnostic methodologies and targeted therapeutic approaches in clinical endodontics. The potential implications extend beyond diagnostic applications to include the development of novel therapeutic strategies targeting specific molecular pathways identified in this study. These findings bridge the gap between basic science and clinical practice, potentially leading to evidence-based molecular diagnostics and therapeutics in endodontics.

The integration of these molecular findings with clinical parameters suggests the possibility of developing personalized treatment approaches based on individual molecular profiles. This paradigm shift in endodontic diagnosis and treatment planning could potentially improve treatment outcomes through more precise, molecularly-guided interventions. Future research directions should focus on validating these molecular signatures in larger populations and developing practical diagnostic tools for clinical implementation.Figs. 3 and 4

## Conclusion

The molecular analysis of irreversible pulpitis demonstrated significant differential expression patterns of multiple inflammatory biomarkers in comparison with noninflamed control specimens. Quantitative examination revealed substantial upregulation of specific molecular markers, including LCP2, PTPRC, CXCL8, TNFα, IL6, CCL2, MMP9, NOD2, ICAM1, and TLR8, in pathologically altered pulp tissue. This cross-sectional exploratory study provides preliminary evidence that distinctive molecular signatures exhibited by these inflammatory mediators present potential diagnostic value in discriminating between pathological and physiological pulpal states. Notably, heightened expression of IL8, TLR8, and TNFα showed positive associations with increased patient-reported pain intensity.

Clinical implementation of these molecular markers necessitates comprehensive validation through large-scale, multicohort investigations correlating molecular profiles with standardized clinical parameters. Future investigative directions should focus on the identification and validation of novel genetic biomarkers utilizing stratified cohorts representing various stages of chronic irreversible pulpitis progression.

The translation of these molecular findings into clinical applications requires several critical developmental phases. Primary considerations include the establishment and validation of reliable biomarker panels capable of accurately reflecting the spectrum of pulpal inflammatory states. Additionally, optimization of specimen collection methodologies, particularly for protein-based biomarkers, requires refinement to ensure consistent yield and analytical reliability. The translation of these molecular markers into point-of-care diagnostic applications faces substantial technological and practical challenges that warrant careful consideration. Current gene expression analysis methodologies, including qRT-PCR and similar molecular techniques, require specialized laboratory equipment, trained personnel, and significant processing time (typically 4-6 hours from sample collection to results), making them incompatible with the time constraints of routine dental practice, where diagnostic decisions are often required within a single appointment. The development of rapid, chair-side platforms necessitates miniaturization of complex molecular assays into portable devices capable of delivering results within 15 to 30 minutes while maintaining analytical sensitivity and specificity comparable to laboratory-based methods. Sample collection from inflamed pulp tissue presents unique challenges, as obtaining adequate quality and quantity of nucleic acids or proteins from small tissue volumes in a clinical setting requires standardized, minimally invasive collection protocols. Economic feasibility represents another critical consideration, as point-of-care devices must achieve cost-effectiveness that justifies their integration into routine practice, with per-test costs competitive with existing diagnostic approaches. Additionally, successful implementation requires development of user-friendly interfaces suitable for general dental practitioners without specialized molecular biology training, comprehensive clinical validation studies demonstrating diagnostic accuracy in real-world settings, regulatory approval pathways through appropriate agencies (FDA, CE marking), and establishment of quality control protocols ensuring consistent performance across diverse clinical environments. These multifaceted challenges highlight the substantial developmental pathway required to translate our laboratory findings into clinically viable diagnostic tools, though recent advances in microfluidics, biosensor technology, and molecular detection systems suggest such translation is increasingly feasible with sustained research and development efforts.

## Author contributions

Raksha Bhat: Conceptualization, methodology, funding acquisition, data acquisition, writing – original draft preparation; Sean Prison D’Souza: Funding, project administration; Preethesh Shetty: Conceptualization, project administration, writing; Shruthi Padavu: Resources, investigation, supervision, writing – review and editing; Praveen Rai: Visualisation, bioinformatic analysis, writing – review and editing; Ballamoole Krishna Kumar: Software, bioinformatics, manuscript revision; Shishir Shetty: Conceptualisation, formal analysis, project administration, writing – review and editing.

## Funding

The financial support for the study was received from Indian Council of Medical Research (ICMR-STS Research grant; 2022-12582).

## Declaration of competing interest

The authors declare that they have no known competing financial interests or personal relationships that could have appeared to influence the work reported in this article.

## Appendix Supplementary materials

Image, application 1

Image, application 2
