# Supplementary material for: Differential Expression Analysis of Key Inflammatory Mediators in Irreversible Pulpitis for Diagnostic Biomarkers
Source: Int Dent J. 2026 May 31;76(4):109596. doi: 10.1016/j.identj.2026.109596 (PMC13231072; doi:10.1016/j.identj.2026.109596)
Supplement: Supplementary file 1 [file mmc1.pdf]

NITTE (Deemed to be University) | A B SHETTY MEMORIAL INSTITUTE OF DENTAL SCIENCES

INSTITUTIONAL ETHICAL COMMITTEE

CHAIRPERSON

Prof. (Dr.) Laxmikanth Chatra
Department of Oral Medicine & Radiology
Yenepoya Dental College, Mangalore

MEMBER SECRETARY

Prof. Dr. Amarshree A Shetty
Associate Dean, Student Welfare
Dept. of Pediatric & Preventive Dentistry.

MEMBERS

Prof. (Dr.) U. S. Krishna Nayak
Principal & Dean

Prof. (Dr.) Chethan Hegde
HOD, Prosthodontics and Crown and Bridge

Prof. (Dr.) Manoj Shetty
HOD, Oral Implantology

Prof. (Dr.) Pushparaj Shetty
HOD, Oral & Maxillofacial Pathology and Oral Medicine

Prof. Dr R. Narayana Charyulu
Vice Principal, Nitte Gulabi Shetty Memorial Institute of Pharmaceutical Sciences

Prof. (Dr.) Vijaya Hegde
HOD, Public Health Dentistry,
A.J. Institute of Dental Sciences

Dr. Aniban Chakraborty
Deputy Director, Nitte University Centre for Science Education and Research

Prof. (Dr.) Audrey Madonna D'cruz
HOD, Public Health Dentistry

Dr. Tripthi Shetty
Reader, Oral & Maxillofacial Surgery

Dr. Vivek Nambiar
Lawyer

Mrs. Judith Shaila Lobo
Regional Operations Manager,
Indian Cancer Society

Mr. Ramdas Aithal
Priest, Karkala

Cert.No. ABSM/EC/269/2022 **Date :- 10th August 2022**

To
Sean Prinson D'souza
Principal Investigator
IIIrd Year BDS
A B Shetty Memorial Institute of Dental Science

Dear Sean Prinson D'souza

Ref.:- **Evaluation of hub genes for diagnosis of irreversible pulpitis ; promoting success of vital pulp therapy.**

At the Ethics Committee meeting held on 9th July 2022 where your study was discussed, the Committee has decided to **Approve and Grant Ethical Clearance** for the study to be carried out by the Principal Investigator at A B Shetty Memorial Institute of Dental Sciences, Deralakatte, Mangalore

Yours Truly,

Prof.Dr. Amarshree A Shetty
Member Secretary,
Institutional Ethical Committee
ABSMIDS.

MEMBER SECRETARY
INSTITUTIONAL ETHICAL COMMITTEE
ABSMIDS

Scanned by TapScanner
