# Supplementary material for: Differential Expression Analysis of Key Inflammatory Mediators in Irreversible Pulpitis for Diagnostic Biomarkers
Source: Int Dent J. 2026 May 31;76(4):109596. doi: 10.1016/j.identj.2026.109596 (PMC13231072; doi:10.1016/j.identj.2026.109596)
Supplement: Supplementary file 2 [file mmc2.pdf]

DEPARTMENT OF CONSERVATIVE DENTISTRY & ENDODONTICS

PATIENT INFORMATION SHEET

Name :

Age:

Address:

Tel no:

Date :

O.P Number:

The information in this document is meant to help you decide whether or not to take part in this study. You are being asked to participate in this study as you satisfy our eligibility criteria. You will be one of those patients we plan to recruit in this study.

Introduction:
You are invited to participate in a research. This document gives you a description ofthe study in which you are being asked to participate. Your participation in this study is voluntary, and you can enquire about all details before giving your written consent to participate in the study.

Purpose:
The purpose of this study is to evaluate the extent of inflammation associated with the affected tooth and to determine measures to help improve the outcomes of teeth indicated for root canal treatment.

Procedure:
The study is conducted on patients indicated for root canal treatment. After administration of local anaesthesia in teeth, pulp tissue will be obtained from the root canal as a part of the procedure to clean and disinfect the root canals for successful treatment. The DNA/RNA extracted from the sample will only be used for research to understand the disease.

Risks:
As the procedure is conducted after administration of local anaesthesia for routine endodontic procedure, the procedure will not cause any sort of pain or discomfort. In the event of any discomfort it will be taken care of by the researcher.

Costs:
The participant will not be charged for this procedure.

Benefits:
Participating in this study will help understand the extent inflammation associated with your affected tooth, helping us improvise on the treatment plan.

Confidentiality:
The information in the study records will be kept confidential. The data will be securely stored and made available only to persons conducting the study and to the regulatory authorities. No reference will be made in oral or written reports which could link you to the study.

Compensation:
In the event of an injury occurring to the participant, such participant shall be provided free medical management as long as required or till such time it is established that the injury is not related to the procedure, whichever is earlier.

Contact:

If you have questions at any time about the study or the procedures, (or you experience adverse effects as a result of participating in this study,) you may contact the researcher, Dr. Raksha Bhat, at Dept. of Conservative Dentistry and Endodontics.

Participation:

Your participation in this study is voluntary; you may decline to participate at any time without penalty and without loss of benefits to which you are otherwise entitled.

If you withdraw from the study prior to its completion, you will receive the usual standard of care for your disease, and your non-participation will not have any adverse effects on your subsequent treatment or relationship with the treating doctor.
If you withdraw from the study before data collection is completed, your data will not be entered in the study report.

Informed consent for participation in the Proforma.

Study title: Differential expression analysis of key inflammatory mediators in irreversible pulpitis: a prospective molecular investigation for novel diagnostic biomarkers

I confirm that I have been explained the study by the researcher/moderator and I had an opportunity to ask questions.

- I have read and understood this consent form and the information provided to me.
- I understand that the participation in the study is voluntary and I am free to withdraw at any time without giving any reason and without being my medical care and legal rights being affected.
- My rights and responsibilities have been explained to me.
- I hereby give permission to the investigator to release information obtained from me as a result of participation in the study to the sponsors, regulatory authorities, government agencies and ethical committee.
- I have understood that my identity will be kept confidential if my data are publicly presented.
- I agree not to restrict the use of any data or results that arise from this study provided such a use is only for scientific purpose(s).
- I have decided to include myself in the study.
- By signing this consent form, I attest my information to this study and my willingness to be a part of this study.

Name and signature of the participant [With Date]

____

Name and signature of the investigator/representative obtaining the consent [With Date]

____

Name and signature of the impartial witness [With Date] (if required)

____

Informed consent for participation in providing Biological sample.

Study title: Differential expression analysis of key inflammatory mediators in irreversible pulpitis: a prospective molecular investigation for novel diagnostic biomarkers

Name :
Age:
Address:
Tel no:
Date :
O.P Number:

Do you consent to biological sample study?

☐ YES, I consent ☐ NO, I do not consent

- I understand that I am being invited to take part in the research study.
- I confirmthat I have read and understood the information sheet dated________for the above study and have had the opportunity to ask questions.
- I understand that my participation in the study is voluntary and that I am free to withdraw at any time, without giving any reason, without my medical care or legal rights being affected.
- I understand the risks and potential benefits of this research study that were explained to me. I freely give my consent to take part in research study described in this form.
- I understand that the Sponsor of the research study, others working on the Sponsor's behalf, IEC and the regulatory authorities will not need my permission to look at my health records both in respect of the current study and any further research that may be conducted in relation to it, even if I withdraw from the trial. I agree to this access. However, I understand that my identity will not be revealed in any information released to third parties or published.
- I agree not to restrict the use of any data or results that arise from this study provided such a use is only for scientific purpose(s).
- I agree to take part in the above study.

Name and signature of the participant [With Date]. ____________________

Name and signature of the investigator/representative obtaining the consent [With Date] ____________________

Name and signature of the impartial witness [With Date] (if required) ____________________

____

In case of any queries, Kindly contact
The Chairperson
Central Ethics Committee
NITTE (Deemed to be university)
Deralakatte, Mangalore
Ph. no: 0824-2204300/01/02
